# 

**Published:** 1852-12

**Authors:** 


### WESTERN JOURNAL

OF

### MEDICINE AND SURGERY.

EDITORS.

---

LUNSFORD P. YANDELL, M. D.

PROFESSOR OF PHYSIOLOGY AND PATHOLOGICAL ANATOMY

IN THE UNIVERSITY OF LOUISVILLE.

AND

THEODORE S. BELL, M.D.

We are pleased to learn that our friends in the Law Department have an unusually large class. Talents, attainments, and efforts to build up a first-rate school, such as theirs, ought to be successful, and it may safely be predicted will be.

---

### OUR NEXT VOLUME.

Our Journal has now been in existence thirteen years. This number completes the 26th volume. It is the oldest medical journal in the West, and has been sustained so long that we cannot think of allowing it to come to an end, but on the contrary are making preparations to carry it on with renewed vigor. With our next issue we shall commence a new series, and hope to appear in a new type and upon paper of a better quality.

Our contributors also promise to give us more aid than they have done for sometime past. We do not like to make large promises, but we think we can assure our readers that they will find an improvement in our future numbers. We pledge ourselves to do all in our power to merit their approbation and patronage.

BOOKS RECEIVED.

"Simon's General Pathology" has been issued by Blanchard & Lea in their usual neat and tasteful style, and a copy of the work has been laid on our table. We shall hasten to give our readers some account of its valuable contents.

We are indebted to Lippincott, Grambo, & Co. for a superb work—the "*Operative Surgery*" of Henry H. Smith, M. D.—a work which, we feel very sure, is destined to take a high rank upon the writings on Surgery. In fulness of detail and elegance of execution it will compare with any volume yet issued from the American medical press.

### INDEX TO VOL. X.

Abscess of Lung..................245
Abortion in the Vomiting of pregnancy..........................542
Acid, Nitric in Hooping Cough...259
Alligator, respiration of..........166
Anesthesia, summary of deaths from.............................. 62
Anesthetics in operations..........251
Asthma, pathology of............ 71
Animal heat..................443, 446
Association, American Med....... 74
Association of Botanists.......... 90
Association, Med., N. Y............331
Association, Med., Missouri......336
Appointments, professional.......364
Arsenic, antidotes to..............152
Auscultation, Flint on..............475

B

Bennett's case of hematemesis....471
Blisters in gleet......................150
Bone, superior maxillary, excision.185
Bone, inferior maxillary, excision..277
Bernard's Surgery....................242
Bite of the Rattlesnake..............255
Bowels, Chloroform in spasm of...258
Bray's case of tumor.................396
Bright, case reflex nervous action.303
Bright on Laryngismus stridlus.....462
Buckler on cholera in Baltimore..138

C

Caldwell on vital chemistry.......103
Cancer, iron in.......................153
Cartwright, Dr. S. A. and his critics362
Cholera in Baltimore...............138
Cholera, Leszczynski on...........369
Cholera and Dysentery..............367
Chloroform, poisonous..............365
" in operations.........251
" as an emmenagoge...158
" causes of death from..166
" in delirium tremens...535
" poisoning from swallowing545
Clinical Reports, Flint's............393
Cantharides in ulcers............. 59
Colica picto num, chloroform in....545
Contagion of Typhoid Fever..68, 88
Conception before menstruation...451
Cooper's Well, analysis of....... 30
Cooper's, Bransby B., surgery....243
Coryza, intermittent.................358
Critic, liberality of ..................355
Croup, chloroform in...............262
Crinoidea in western states....... 79
Cutaneous diseases, starch in .....268

D

Darby on water in disease........ 1
Delirium tremens, chloroform in...535
Diarrhœa, sulphuric acid in........538
Dowler, Tableaux of N. O......... 34
Drake, Dr., death of................548
Durrett on Dysentery............... 17
" on fracture of head....... 99
Dysentery in L. M. Hospital..... 17
" Sulphuric Acid in.....538
" and Cholera..........367

E

Emmenagogue, chloroform.........158
Epidemics, summer.................. 249
Epilepsy, tracheotomy in...........263
Ergot of rye, its action............ 65
Excision of lower jaw...............185
" of upper jaw..............277
" of large tumor...........396
Eye destroyed by a bird-shot......250

F

Fellis Bovini as a medicine.......429
Fever, continued.........88, 273, 398
" opium large doses in...... 60
" quinine in.............86, 164
" relapsing ....................353
" yellow........................238
Flint on Continued Fever....273, 398
" on valvular diseases of heart 141
" on percussion and auscultation ..............................475
Fractures, Knapp on............... 18
Fracture of arm from muscular contraction .........................261
Fishback, spinal irritation ........ 93

G

Georgia, quackery in...............183
Glanders in human subject.........101
Gleet, blistering in ..................150
Grant on health of Memphis.......134
Green on polypi of larynx.........239
Grimes' Dr., Address ...............181
Gross on Extirpation Maxillæ.185, 278

H

Hair, habit of swallowing.........156
Head, injuries of.................. 99

Heat, animal............443, 446, 448
Heart, valvular diseases of........141
Hernia, radical cure of..........433
" in a young child..........438
" new mode of reducing....267
Homœopathy...................359
Hooping Cough.............259, 437
Huette's surgery................252
Hydrophobia, proportion of cases..539
Hydrocephalus .................452
Hydrophobia.....................162
I
Injection for cure of Hernia.......433
Insane, improved condition of.....358
" muscular power of.......357
Injury friendly, suit for............532
Intermittent coryza................258
Itermittent fever and table-salt....252
Iron, lactate, in cancer............153
Iron, peroxide, arsenic.............152
Itch, cured in two hours........... 58
J
Jaw, excision of.............185, 277
K
Kentucky Medical Society...461, 457
Knapp on Fractures ............. 18
L
Lactate of iron in cancer..........153
Leszczynski on cholera............360
Lesions of heart .................141
Lung, abscess of...................245
Larynx, polypi of.................239
M
Manganese in anemic diseases....533
Medicine, Wood's Practice of....229
Memphis, health of...............134
Menstruation and disease.........356
Muscles, nutrition of..............160
Miller's Surgery...................243
N
New Orleans, tableaux of......... 34
Nervous action, reflex............303
O
Obituary.....................368, 450
Obstruction of bowels...........258
Oedema of glottis................238
Opium in fevers................. 60
Operations on the jaw......186, 278
Operation for Hernia.............438
" of Tracheotomy........263
P
Paternity, doubtful................308
Phlegmasia, dolens...............248
Pirrie's Surgery...................243
Pitch, variations of in percussion..475
Poisoning, oil Tansy...............440
" on temperature.........443
Pumpkin seed in tenia............435
Pure medicines...................269
Q
Quackery in Georgia.............183
Quinine in continued fever........164
R
Ramsey's Record of Medicine.... 48
Rappers, Spiritual................ 89
Rattlesnake bite of...............255
Report of Faculty University of Pa.417
Respiration of Alligator..........166
Rheumatism.....................260
Rye, ergot of.................... 65
S
Salivation, prevention of.........439
Smith's analysis Cooper's Well.... 30
Snake, bite of....................255
Sodium chloride, ague............252
Springs Mineral...................532
Starch in cutaneous diseases.......258
Stone in bladder and sulphur water272
Sulphuric acid in diarrhœa.........538
Surgery, Bernard and Huette's...242
" B. Cooper's, and Pirrie's.243
" Miller's.....................243
Sulphur water in stone...........272
Sutton's case.....................308
Syringe, improved................ 360
T
Tableaux of New Orleans........
Tænia, pumpkin seeds in..........435
Talcott's Address.................140
Tansy, poisoning by..............440
Tincture cantharides.............. 59
Tumor, Bray's case...............396
Typhoid fever................68, 88
Typhus.......................... 86
Transylvania Med. Jour...........367
U
Ulcers, Tinct. Cantharides........ 59
University of Louisville..91, 269, 361
" of Nashville.......... 92
" of Pennsylvania......417
V
Veratrum viride.............49, 56
Vomiting in pregnant women.... 542
W
Warts, cancerous degeneration.... 339
Wood's Practice..................229
Wounds, gunshot.................251
Y
Yandell on the Crinoidea..........79

### *Receipts of Medical Journal for November* 1852.

| | |
|---|---|
| Dr. T. G. Moore, Harrodsburg, Ky., - - - - | $5 00 |
| Dr. Seaton, Jeffersontown, Ky., - - - - - | 10 00 |
| Dr. E. D. Foree, Williamson, Ky., - - - - | 5 00 |
| Dr. Gregory, La Grange, Texas, - - - - - | 4 50 |

### TO OUR SUBSCRIBERS.

The end of the year is approaceing, when all punctual men pay their debts. Gentlemen, you are collecting your dues now, and let us hope you will not forget the publishers of your Medical Journal. We cannot go on without money. If you expect us to work for your gratification and instruction, you must pay us for it. We appeal to your *liberality*—shall we say? No. To your sense of justice.

PRENTICE & HENDERSON.

Dec. 1852.

Dec. 1852—m1

---

The Western Journal of Medicine and Surgery is published monthly by the undersigned, at the corner of Main and Fifth streets, Louisville, at $5 per annum, payable in advance. Each number contains 96 pages, making two volumes in the year of more than 550 pages each.

Contributions to its pages by the Physicians of the Valley of the Mississippi addressed to the Editors, are respectfully solicited.

Letters on business, to be addressed, postage paid, to the publishers.

PRENTICE & HENDERSON

